# A20 haploinsufficiency in a neonate caused by a large deletion on chromosome 6q

**DOI:** 10.1186/s12969-023-00947-z

**Published:** 2024-01-05

**Authors:** Fan Zhang, Liang Zhang

**Affiliations:** 1 Department of Neonatology, Hunan Children’s Hospital, Changsha, Hunan China; 2 Department of Nephrology, Rheumatology and Immunology, Hunan Provincial Key Laboratory of Pediatric Orthopedics, Hunan Children’s Hospital, Changsha, Hunan China

**Keywords:** A20 haploinsufficiency, Chromosome deletion, Autoinflammatory, Neonate

## Abstract

Haploinsufficiency of A20 (HA20) is a rare monogenic disease caused by heterozygous loss-of-function mutations in the tumor necrosis factor alpha-induced protein 3 (*TNFAIP3*) gene located on chromosome 6q23.3. The majority of disease-causing mutations in most cases of HA20 comprise single nucleotide variations, small insertions, or deletions in *TNFAIP3,* which result in a premature termination codon and subsequent disruption of its anti-inflammatory role. Large deletions have been reported sporadically. HA20 patients may present with a variety of autoinflammatory and autoimmune features during early childhood; however, cases with neonatal onset are rare. Here, we describe a Chinese neonate presenting with concomitant inflammatory and other syndromic manifestations caused by a 5.15 Mb interstitial deletion in chromosome 6; these deletions affect *TNFAIP3*. Taken together, the data extend the clinical and genetic spectra of HA20.

## Background

Heterozygous pathogenic mutations in the gene encoding tumor necrosis factor alpha-induced protein 3 (*TNFAIP3*) result in haploinsufficiency of A20 (HA20), a rare hereditary autoinflammatory disease [1–3]. The *TNFAIP3* gene, located on chromosome 6q23.3, encodes the A20 protein, which acts as a central gatekeeper for negative regulation of NF-κB signaling, NLRP3 inflammasome activity, and apoptosis [4, 5]. HA20 is characterized typically by various autoinflammatory features that resemble Behçet’s disease, or by autoimmune symptoms that mimic systemic lupus erythematosus, or correlated with allergy-associated features [1, 6–9]. So far, more than one hundred HA20 patients have been reported in the literature, and the majority of TNFAIP3 variants are monoallelic nonsense and frameshift mutations, small insertions, or small deletions; the disease commonly manifests in early childhood [10–12]. However, cases with neonatal onset and/or caused by copy number variations resulting from large contiguous gene deletions of 6q involving the TNFAIP3 gene are rare. Here, we described a neonate presenting with concomitant inflammatory and other syndromic manifestations caused by a 5.15 Mb interstitial deletion on chromosome 6*.*

## Clinical report

**Fig. 1:**
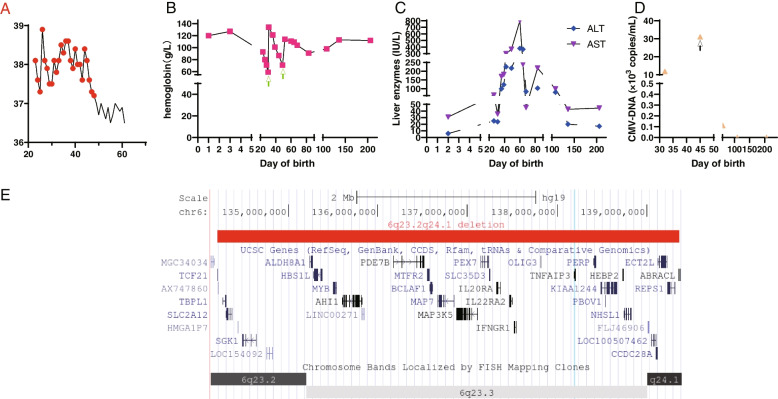
Clinical manifestations and genetic characteristics. **A** Daily maximum core temperature in the neonate from Day 23 post-birth. **B** Hemoglobin (g/L) levels measured during febrile episodes, as well as basal levels measured at birth and follow-up (green arrows denote infusions of hemoglobin). **C** Circulating concentrations of alanine transaminase (ALT; IU/L, blue quadrangle) and aspartate transaminase (AST; IU/L, purple triangle) over time. **D** Fluctuation of blood serum CMV-DNA levels (10^3^ copies/mL); the black arrow indicates initiation of ganciclovir treatment. **E** A large deletion in chromosome 6 and OMIM genes encompassing the 6q23.2q24.3 region

The neonate was born to a 29-year-old mother via in vitro fertilization. She was preterm (delivered, at a gestational age of 36 weeks, by cesarean section) and weighed 2700 g. There was no consanguinity, significant family history of immunodeficiency, or abnormalities detected during pregnancy. She presented with decreased activity and clear growth delay. Since the age of 23 days, she suffered from recurrent episodes of low to moderate-grade fever (37.6–39℃) one to three times per day. She was treated preliminarily with antibiotics, including amoxicillin/potassium clavulanate followed by cefotaxime for two weeks, but the febrile attack did not subside immediately; rather, it resolved gradually within 24 days (Fig. 1a).

On admission, extrauterine growth restricted was noted. Body weight and head circumference were below the 3rd and 10th percentiles, respectively. Physical examination revealed enlarged liver and spleen, 4 cm below the right costal margin and 2.5 cm below the left costal margin, respectively. Blood analysis revealed slightly elevated white blood cell counts, and C-reactive protein levels, which gradually returned to normal levels accompanied by extinguished fever within 22 days. She suffered worsening of her acute severe anemia with elevated percent of reticulocyte up to 3.87% gradually after the onset of fever (Fig. 1b). However, there was no clear evidence of hemoglobin loss or hemolysis, without clinical signs of active bleeding or jaundice; routine test of urine, feces and kidney function were normal without hyperbilirubinemia or increased serum lactate dehydrogenase; direct antiglobulin test, hemoglobin analysis, red cell osmotic fragility test and enzymatic activity test of G6PD were negative. Liver enzymes were elevated constantly for 10 days after onset of the febrile attacks (Fig. 1c). No immunosuppressive treatment rather than regular project therapy was given, however, the ameliorative level of liver enzymes and anemia was correlated with gradual resolution of febrile attack. Mild anemia and hepatic injury reappeared after another slight fever lasting for about one week (Fig. 1b, c).

Measurement of autoantibodies, anti-toxoplasma gondii, Rubella Virus, Cytomegalovirus, and Herpes simplex virus (TORCH) were normal. Pathogen cultures were negative, as well as tandem mass spectrometry and gas chromatography-mass spectrometry for screening particular inheritance metabolism diseases. No other obvious abnormalities were found in ophthalmologic assessment, hearing test and brain MRI scans. Abdominal ultrasonography showed no other abnormalitis except for hepatomegaly.

Metagenomic next generation sequencing was performed to detect pathogenic agents, which showed CMV positive, and PCR detected cytomegalovirus (CMV) DNA in the serum, she was given ganciclovir for two weeks (Fig. 1d). An unexpected discovery was that the metagenomic next generation sequencing simultaneously prompted genomic deletions. In light of this surprise tip, analysis of copy number variations were performed using a comparative genomic hybridization array and revealed a heterozygous 5.15 Mb deletion in 6q23.3q24.3 (Fig. 1e). The deletion affected at least 30 genes, including *TNFAIP3*. Finally, she was definitively diagnosed with HA20.

She subsequently experienced developmental delay in motor, and also presented growth retardation since the age of six months, at that time, ameliorated hepatomegaly was revealed as enlarged liver was 3 cm below the right costal margin, and her spleen was not palpable. The COVID-19 epidemic led to suspension of her out-patient clinics and follow-up visits.

## Literature review

An English language search of the PubMed database was conducted up until 31st August 2023; the aim was to identify studies reporting clinical features of patients with HA20 caused by contiguous deletions of 6q including TNFAIP3.

## Results

**Fig. 2:**
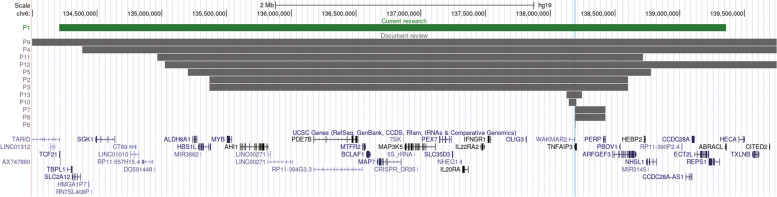
Schematic representation of pathogenic deletions in chromosome 6 affectingTNFAIP3

At present, only 11 patients with HA20 molecularly confirmed to be caused by deletion of chromosome 6q have been reported since 2016 [13–20]. The initial clinical characteristics and genetic evaluation of all 12 patients are summarized in Table 1 and Fig. 2. Of all patients, eigtht were female, and five presented with de novo deletions on chromosome 6q. Nine patients experienced early-onset symptoms before the age of 1 year old; in seven patients, the initial symptoms were unprovoked and recurrent episodes of fever. Additionally, dysmorphic features and intellectual disability were reported sporadically, contributing to the complex phenotype. Intriguingly, it was assumed that the symptoms of HA20 may depend on genetic background and environmental factors; indeed, patients with A20 mutations widely distributed from the OTU to the ZnF7 domains from East Asia presented more frequently with recurrent fever [12]. Correspondingly, to some extent, unprovoked and recurrent episodes of fever as the initial symptoms were slightly more frequently observed in these patients from East Asia harboring deletion of 6q involving the TNFAIP3 gene. More cases are warranted to consolidate this phenomenon.

**Table 1:** Demographic data and initial features of patients with deletions on chromosome 6q in the TNFAIP3 gene

	Current report	*Endo et al.*		*Franco-Jarava et al.*	*Sun et al*	*Taniguchi et al.*	*Tsuchida et al.*		*Viel et al.*	*Wan et al.*	*Wu et al.*	
	*Patient 1*	*Patient 2*	*Patient 3*	*Patient 4*	*Patient 5*	*Patient 6*	*Patient 7*	*Patient 8*	*Patient 9*	*Patient 10*	*Patient 11*	*Patient 12*
Ethnicity	Chinese	Japanese	Japanese	Spanish	Chinese	Japanese	Japanese	Japanese	French	unknown	Caucasian	African American
Gender	Female	Female	Male	Male	Female	Female	Female	Female	Male	Female	Female	Male
Inheritance	de novo	paternal	unknown	de novo	de novo	de novo	maternal	unknown	unknown	unknown	de novo	unknown
Size of deletion	5.15 Mb	3.3 Mb	3.3 Mb	13.13 Mb	3.576 Mb	0.119 Mb	0.236 Mb	0.236 Mb	5.5 Mb	0.0599 Mb	3.4 Mb	11.7 Mb
Age of onset	23 days	6 months	1 year	7 months	2 months	3 weeks	2 months	1 year	5 years	35 years	6 months	3 years
Age at diagnosis	2 months	Not mentioned	Not mentioned	12 years	45 months	8 months	11 years	16 years	19 years	56 years	6 years	7 years
Initial symptoms	Fever	Fever AIHA	AIHA	Fever Respiratory- infections Gastroenteritis	Fever	Fever	Fever	Oral ulcers	Henoch-Schönlein purpura	Allergy Rosacea	Fever Infections Oral ulcers	Oral ulcers
Fever	+	+	-	+	+	+	+	-	-	-	+	-
Lymphadenopathy	-	+	+	+	+	-	+	+	-	+	+	-
Hepatomegaly	+	+	+	+	+	+	-	-	-	-	+	-
Splenomegaly	+	+	+	+	+	+	-	-	-	+	+	-
Anemia	+	AIHA	AIHA	-	HLH	-	-	-	AIHA	-	-	AIHA
Hepatic dysfunction	+	-	-	-	-	-	-	-	-	-	-	-
Failure to thrive	+	Not mentioned	Not mentioned	+	+	+	+	Not mentioned	+	Not mentioned	+	+
Oral ulcer	-	-	-	+	-	+	+	+	-	-	+	+
Arthralgia	-	-	-	-	-	+	+	-	+	-	-	-

*AIHA* Autoimmune hemolytic anemia, *HLH* Hemophagocytic lymphohistiocytosis

## Discussion

In this report, we presented a Chinese HA20 neonate presenting with concomitant inflammatory and other syndromic manifestations caused by a 5.15 Mb interstitial deletion in chromosome 6. We also reviewed the literature to include cases of patients with HA20 caused by contiguous deletions of 6q including TNFAIP3. In total, 12 cases have been reported to date. Most of the patients were from East Asia and presented with recurrent fever before the age of 1 year old. It has been considered that diagnosis of HA20 is challenging due to its heterogeneous clinical presentation and the lack of pathognomonic symptoms, especially in neonates and infants. Recurrent stomatitis is the most common symptom in HA20 patients caused by single nucleotide variations, small insertions, or small deletions in TNFAIP3. In contrast, our result and other literature show that in HA20 patients resulting from contiguous deletions of 6q including TNFAIP3, unprovoked and recurrent episodes of fever tends to be the most common symptom. Thus, HA20 should not be neglected and should be considered in the differential diagnosis of newborns and infants with recurrent episodes of fever.

The zinc finger protein A20, a unique and potent regulator of ubiquitin (Ub)-dependent signaling encoded by the *TNFAIP3* gene located on chromosome 6q23.3, plays a vital role in the immune homeostasis. A20 functions as a ubiquitin-editing enzyme (deubiquitinase [DUB]) that inhibits key proinflammatory molecules and protects cells from death [21]. This is attributed mainly to its role in modulating nuclear factor kappa B (NF-kB) signaling cascades as part of its key function as a negative regulator of inflammation and immune responses. A20 inhibits not only tumor necrosis factor (TNF)-dependent activation of nuclear factor (NF)-kB, but also the activation of NF-kB in response to interleukin (IL)-1; it also inhibits signaling cascades mediated through pattern recognition receptors (PRRs) and T cell and B cell antigen receptors; These cascades control crucial stages of immune cell homeostasis, including apoptosis, necroptosis, and inflammasome activity [22–24].

Thus, A20 is a potent anti-inflammatory molecule. Indeed, mice harboring targeted cell-specific deletions in the *TNFAIP3* gene in innate immune cells develop autoinflammatory diseases spontaneously, and mice harboring targeted cell-specific deletions in adaptive immune cells develop spontaneous inflammation that resembles human autoimmune diseases [24]. Actually, A20 has attracted attention due to its multiple links to a variety of human diseases. Single nucleotide polymorphisms (SNPs) in the A20 gene locus, which reduce expression of A20, were identified initially as putative risk alleles for a range of inflammatory and autoimmune pathologies, including rheumatoid arthritis, inflammatory bowel disease, and systemic lupus erythematosus [25].

In 2016, a new autoinflammatory disease presenting as an early-onset autoinflammatory condition resembling Behçet’s disease (BD), and caused by heterozygous loss-of-function mutations in the *TNFAIP3* gene, was described and named A20 haploinsufficiency (HA20) [1]. Since then, numerous cases with a broad spectrum of clinical presentations associated with autoinflammatory syndromes, autoimmune diseases, and immunodeficiency have been reported worldwide. To date, more than one hundred HA20 patients have been reported in the literature, particularly in East Asian countries such China and Japan [10–12, 26]. The majority of TNFAIP3 variants harbor monoallelic nonsense and frameshift mutations, small insertions, and small deletions, leading to a premature termination codon and disrupted anti-inflammatory effects [10–12]. Overall, HA20 patients caused by copy number variations resulting from contiguous deletions of 6q (involving the *TNFAIP3* gene) are rare [13–20]. Correspondingly, in clinical practice, the major phenotypes of HA20 caused by truncating and missense mutations are Behçet’s disease-like symptoms, with recurrent oral ulcers as the primary incipient sign, or autoimmune-like symptoms that mimic systemic lupus erythematosus, autoimmune thyroid disease, type 1 diabetes, autoimmune hemolytic anemia (AIHA), and autoimmune hepatitis [12]. Until now, systematic correlation between the contiguous deletions of 6q and disease diagnosis has been far unavailable, actually, the diagnosis and management tends to be complicated in those caused by copy number variants involving the *TNFAIP3* gene [19].

The neonate in our study initially presented with unprovoked and long-lerm fever, associated with no apparently infectious manifestation, furthermore, adequate antibiotics therapy was administered without significant efficiency against the fever. It appeared that the fever was not attributed to the CMV infection as the resolution of fever had revealed before ganciclovir therapy initiation.

It is also established that A20 is a crucial hepatoprotective factor in mice; indeed, A20 knockout resulted in excessive multi-organ inflammation (including the liver) [5]. Moreover, hepatocyte-specific A20 knockout mice showed sustained activation of NF-κB-dependent genes and increased apoptosis of hepatocytes, which was associated with liver failure [27, 28]. Additionally, dendritic cells-specific A20 knockout mice resulted in liver pathology characterized by inflammatory infiltrates adjacent to the portal triads [29]. As a consequence, elevated liver enzymes have been reported in HA20 patients with mutations in either the ZnF- or OTU-coding regions [10, 30].

Our patient is the first reported case of a large deletion of 6q to present with liver injury. Furthermore, anemia caused by iron deficiency and AIHA has been reported in patients with HA20, which was not in accordance with our patient. Notably, A20 Hem-KO mice develop anemia due to a striking reduction of erythropoiesis in the bone marrow mediated pathophysiologically by IFN-γ [31, 32], which could partly interpret acute anemia in our patient.

As mentioned above, HA20 caused by this large deletion in 6q is likely responsible for the recurrent fever and continuous liver injury, as well as acute anemia and hepatosplenomegaly, notwithstanding that CMV infection might complicate matters.

## Conclusion

Here, we describe for the first time a neonate presenting with concomitant complicated features caused by a large genomic deletion on chromosome 6q.

## Data Availability

The authors declare that all data supporting the findings of this study are available within the article.

## Abbreviations

HA20: Haploinsufciency of A20
TNFAIP3: Tumor necrosis factor alpha-induced protein 3
NF-κB: Nuclear factor-κB
NLRP3: NOD-like receptor thermal protein domain associated protein 3
G6PD: Glucose-6-phosphate dehydrogenase
MRI: Magnetic resonance imaging
TORCH: Toxoplasma gondii, other, rubella virus, cytomegalovirus, herpes simplex virus
CMV: Cytomegalovirus
AIHA: Autoimmune hemolytic anemia
